# Use of high throughput DNA analysis to characterize the nodule-associated bacterial community from four ages of *Inga punctata* trees in a Costa Rican cloud forest

**DOI:** 10.3934/microbiol.2024027

**Published:** 2024-07-23

**Authors:** William D. Eaton, Debra A. Hamilton, Wen Chen, Alexander Lemenze, Patricia Soteropoulos

**Affiliations:** 1 Biology Department, Pace University, One Pace Plaza, New York, NY 10038, U.S.A; 2 University for Peace, El Rodeo de Mora, San José, Cd Colón, Costa Rica; 3 Vermont Cooperative Fish and Wildlife Research Unit, Rubenstein School of the Environment and Natural Resources, University of Vermont, Burlington, VT 05405, U.S.A; 4 Monteverde Institute, Puntarenas, Costa Rica; 5 Molecular and Genomics Informatics Core (MaGIC), Rutgers New Jersey Medical School, 205 South Orange Avenue, Newark, NJ 07103, U.S.A; 6 The Genomics Center and Dept of Microbiology, Biochemistry and Molecular Genetics, Rutgers New Jersey Medical School, 185 South Orange Avenue, MSB F653, Newark, NJ 07103, U.S.A

**Keywords:** root nodule bacteria, root nodule-associated bacterial community, *Inga punctata* root nodules, plant growth-promoting bacteria, *Bradyrhizobium diazoefficiens*, DNA analysis of root nodule bacteria

## Abstract

Leguminous tree root nodule nitrogen-fixing bacteria are critical for recuperation of soil C and N cycle processes after disturbance in tropical forests, while other nodule-associated bacteria (NAB) may enhance nodule development and activity, and plant growth. However, little is known of these root nodule microbiomes. Through DNA analysis, we evaluated the bacterial taxa associated with the root nodules of the 1-year-old, 2-year-old, 13-year-old, and old growth *Inga punctata* trees in a cloud forest. *Bradyrhizobium diazoefficiens* was the dominant taxon found in all nodules at 63.16% to 85.71% mean percent sequences (MPS) of the total nodule bacterial DNA and was found in the youngest nodules examined (1 year old), suggesting that it is the primary nodular bacteria. There were 26 other NAB genera with collective MPS levels between 7.4% to 12.2%, while 15 of these genera were found in the Bulk Forest soils at collective MPS levels of 4.6%. These bacterial community compositions were different between the NAB and Bulk Forest soils, suggesting the NAB became concentrated within the root nodules, resulting in communities with different compositions from the Bulk Forest soils. Twenty-three of the 26 NAB genera were previously identified with the potential to perform 9 plant growth promoting (PGP) activities, suggesting their importance in root nodule development and plant growth. These NAB communities appeared to successionally develop over time into more complex taxonomic communities, which is consistent with the outcome of advanced microbial communities following succession. The presence of both *B. diazoefficiens* and the NAB communities in the nodules across all ages of tree roots, and the potential for PGP activities linked with most of the NAB genera, suggest the importance of *B. diazoefficiens* and the NAB community for nodule development and enhanced development and growth of *I. punctata* throughout its lifespan, and most critically in the younger plants.

## 1. Introduction

Leguminous trees are known to be an ecologically important species in Central and South American tropical forests, considered critical for tropical forest recovery following deforestation and other disturbances [1]–[3]. Specifically, the leguminous tree root nodule N-fixing bacterial activities provide the principal pathways by which damaged tropical lands recuperate soil N and C lost following deforestation and subsequent agricultural use [1],[2],[4]–[7]. The ecological importance of these trees has stimulated some work to determine the taxonomic identity of the root nodule-forming bacteria from some tropical forest trees [8]–[13], however more work is needed in this area. In addition, it appears that the root nodule bacteria are not alone in the nodule microbiome as it has been recently recognized that there are entire communities of bacteria within these root nodules, known as nodule-associated bacteria, or NAB [14]–[16]. Many of these NAB have plant growth promoting (PGP) metabolic activities, such as N-fixation, pathogen suppression, production of phytohormones for growth, nutrient acquisition, and enhancement of root and shoot length, all of which aid in the primary root nodule development and plant growth [15]–[22]. However, little is known about the NAB communities in tropical forest leguminous tree nodules.

Species of the genus *Inga* are ecologically important leguminous forest trees commonly found throughout the tropical Americas that are thought to enhance the quantity and quality of the soil organic C and N, and stimulate C sequestration and long-term N accumulation [1],[11],[23],[24]. These characteristics, along with *Inga*'s ability to thrive in the high acid soils of the tropical Americas have resulted in its use in tropical reforestation practices in Central and South America [1],[24]. Some work has been conducted to identify the primary root nodule-forming bacteria in several *Inga* species [8]–[11],[25]. As well, several studies had provided an initial assessment of the influence that some *Inga* spp. have on soil ecosystems in Costa Rican forests. In a Northern Zone Costa Rican Forest, *I. edulis* was shown to facilitate the development of a soil microbiome that enhanced soil NH_4_^+^ levels and was associated with a switching of the dominance of 10 functionally redundant N-cycle bacteria taxa in response to disturbance [26]. In the Cloud Forest region of Monteverde, *I. punctata* that is used in forest restoration activities has been found to enhance the taxonomic complexity of its tree-soil N-fixer and lignin-degrader communities, accumulate tree soil C and N, and support land recovery [23]. However, there is still a gap in knowledge of the taxonomic composition of the root nodule or the NAB microbiome of these trees. Such information could help understand how this microbial community influences tree development and growth, if this microbiome composition changes with disturbances (such as deforestation, reforestation, or changing climate), and how such changes might influence tree development and growth or tropical forest ecosystem recovery after perturbations.

Part of the reason for the gap in knowledge is that, often, the taxonomic identity of the root nodule and/or nodule-associated symbionts uses traditional culture methods to isolate the bacteria, which are then taxonomically identified by DNA analysis. However, this method only identifies the nodule bacteria or NAB bacteria that are chemoheterotrophic and capable of being cultured on agar media. This likely misses a majority of the bacterial taxa within the nodule as it is known that the vast majority of bacteria on the planet are either unculturable or have not been cultured to date [27]–[29]. Thus, requiring the ability of any nodular bacteria to be cultured results in an underestimation of the actual number of bacterial taxa associated with the root nodules [30]–[33]. Recently, metagenomic studies have been conducted using DNA samples extracted from disinfected whole root nodules, which are sequenced following PCR amplification, and the taxonomic identity determined using existing databases. These methods have resulted in identification of a much greater diversity of rhizobial and non-rhizobial taxa within root nodules than have been previously identified using culture-based methods [30]–[32],[34],[35]. This culture-independent high-throughput DNA sequencing approach is also more useful in providing information on the relative abundance and distribution of the different rhizobial and non-rhizobial taxonomic populations associated with the root nodules, which cannot be achieved by culture-based methods [30]–[33]. Thus, this approach of genetically identifying the root nodule-associated bacteria via metagenomics can add valuable information concerning the characterization of these microbiome communities that are critically important to the soil ecosystems, and the recuperation of the C and N cycle dynamics in tropical forests after damage.

In the current study, we used an amplicon sequencing analysis of root nodule total DNA to: 1) provide preliminary information on the presumptive identification of the primary nodular bacteria and other NAB taxa in root nodules collected from 1-year-old, 2-year-old, 13-year-old, and old growth *I. punctata* trees all within the same reforestation zone; 2) determine if any of the microbiome taxa had been previously identified as having the potential for plant growth promoting (PGP) activities; 3) determine the earliest age that root nodules could be seen in *I. punctata*; and 4) determine if the overall nodule or NAB microbiome community compositional characteristics differed by tree age.

## 2. Materials and methods

### 2.1. Study site

The *Inga punctata* trees for this study were located in the nursery, restoration sites, and 60+-year-old secondary forest within the Finca Rodríguez Ecological Reserve (FRER), located in Monteverde, Costa Rica (10° 18′ 55.3″ N, 84° 50′ 29.8″ W), at an elevation of between 1250–1280 m asl. This site is within the Premontane Wet Life Zone [36], which is typified as evergreen forest with few deciduous species and moderate epiphyte load [37], with annual precipitation of 2000–2900 mm [38]. The different tree ages used were 1-year-old trees that were germinated from seeds at the nursery in plastic bags with local soil and had not been planted yet (IP1yr samples), 2-year-old trees that were planted as seedlings (IP2yr samples) and had been in the forest for about 1 year, 13-year-old trees also planted as seedlings in the forest (IP13yr samples), and adult trees within the old secondary forest (IPOG). For comparisons to the bacterial community in the forest soils not associated with the root nodules, Bulk Forest soil samples were collected from the old secondary forest for a comparison of the forest soil microbiome to the root nodule microbiomes.

### 2.2. Soil and root nodule collection and preparation

Five *I. punctata* trees from each of the four age classes were chosen within the FRER for the study. Small horizontal tree roots were exposed *in situ* and examined for nodules, with 10 nodules being collected from each of the five trees per age group. Disinfection of all tools and gloves was done between trees pooled by each individual tree. The nodules from each tree were stored in sterile plastic bags for no more than 5 days at 5 °C. To compare the forest soil to the root nodule bacterial community, five 100 m^2^ plots were established within the FRER old secondary forest, and 9 soil cores were collected from each plot using a 7.5 cm × 15 cm × 1.25 cm soil profiler. No forest soil sample was collected within 5 m of the dripline of an *I. punctata* tree to minimize the influence the tree species would have on the bacterial community in the bulk soil sample. All gloves and tools were disinfected initially and then between soil plots to ensure no cross-contamination occurred. The 9 cores from a plot were combined into a sterile bag, providing 5 composite and independent replicate soil samples from the original old secondary forest and are referred to as the forest soil samples.

We expanded on the root nodule sterilization methods of previous authors [30]–[33] to minimize the risk of extracting DNA that was not from endophytic root nodule bacteria. The nodules were rinsed 5 times in sterile water with scrubbing using a sterile soft bristle brush for 1 minute, with water changes each time, then rinsed 5 times in 70% ethanol with scrubbing for 1 minute, with changing of the ethanol with each wash. The nodules were washed 5 times in 4% sodium hypochlorite, with scrubbing, and changing of the solution with each wash, then washed 3 times in hydrogen peroxide with changing of the hydrogen peroxide solution each time, then rinsed 5 times in 70% ethanol with changing of the ethanol, then finally rinsed 5 times in sterile distilled water, with changing of the water each time. After surface sterilization, the nodules were placed on tryptic soy agar plates and incubated for 24 hours to test for levels of sterilization (no bacterial colonies growing), after which the nodules were diced with sterile razor blades, mixed with 150 µL sterile water, and crushed with sterile forceps, then mixed to generate a macerated root nodule sample for each tree for DNA extraction.

An extremely rigorous root nodule surface disinfection process was used to minimize root nodule surface bacteria and the nodules were also incubated on agar plates to ensure no growth of bacteria from their surface to increase the chance of extracting and analyzing only DNA from endophytic bacteria within the root nodule. Nonetheless, as we could not formally discriminate between the DNA analyzed that could have been from a few bacteria embedded on the nodule surface, attached to the inner nodule wall, or as part of the internal nodular tissue, like Hartmann et al. [32], we refer to the microbiome bacterial communities only as being root nodule-associated bacteria (NAB) for this study. However, we also compared the forest soil sample microbiomes to that of the root nodule-associated microbiomes to determine if there were clear differences between these two types of communities.

### 2.3. DNA extraction, sequencing, and bioinformatics

The DNA was individually extracted from each 150 µL macerated root nodule sample and from three 0.33 g replicate forest soil samples using the DNeasy PowerSoil Pro Kit (Qiagen LLC, MD). A NanoDrop 1000 spectrophotometer (ThermoFisher Scientific, Waltham, MA) was used to determine the concentration and purity (A260/A280 ratio) of extracted root nodule DNA prior to downstream analyses. All PCR, DNA sequencing, taxonomic identification methods have been described in detail by McGee et al. [39], involving amplification of the DNA extracts by 2-step PCR reactions, targeting v3 and v4 of the 16S ribosomal RNA gene region for bacteria, with the resulting amplicons being sequenced in Illumina MiSeq runs using a V3 MiSeq sequencing kit (FC-131-1002 and MS-102-3003). The resulting 16S sequences amplicon sequence variants (ASVs) were identified and assigned taxonomic classifications utilizing dada2 (https://www.ncbi.nlm.nih.gov/pmc/articles/PMC4927377/) from phylum to species based on the SILVA rRNA database (build 138) for bacteria [40]. All DNA sequence data have been submitted to the NCBI-Sequence Read Archive (SRA) repository with SRA Project Code PRJNA1019742. The number of times a specific DNA-identified genus appeared in a sample was converted to the mean percent of the sequences (MPS) per sample, as per Weiss et al. [41].

To conduct a preliminary assessment of the potential PGP capabilities of the NAB, the literature (see Table S1) was used to identify genera with MPS levels > 0.1% that had previously been shown to commonly be associated with having the potential for the PGP activities of protease, lipase, or cellulase activity, PO_4_^3−^ solubilization, CO_2_/C1 fixation, IAA (the growth hormone indole-3-acetic acid) production, or siderophore production. In addition, the NAB genera with MPS > 0.1% were also analyzed to determine if they had been previously identified as having the potential for N-fixation or ammonium oxidation (AMO) activity. To do this, these genera were compared to those found with the N-fixation or AMO activity as identified within the KEGG database (https://www.genome.jp/kegg/), the Ribosomal Database Project (RDP) Classifier (https://sourceforge.net/projects/rdp-classifier/), the FAPROTAX database (http://www.loucalab.com/archive/FAPROTAX/lib/php/index.php?section=Download), lists from the International Committee for Systematics of Prokaryotes, Subcommittee on the Taxonomy of Rhizobia and Agrobacteria (http://edzna.ccg.unam.mx/rhizobial-taxonomy/), Weir [42] and the current taxonomy of rhizobia (https://www.rhizobia.co.nz/taxonomy/rhizobia), and 10 different literature sources (see Table S2).

### 2.4. Data analysis

#### 2.4.1. Differences in MPS of bacterial taxa associated with the root nodules

The MPS values of all bacterial genera associated with the root nodules and in the Bulk Forest soils were calculated. The DNA sequences of any bacterial genera associated with the root nodules that were clearly dominant within the root nodules but not common in the Bulk Forest soils were further assessed by NCBI BLAST searches to make a preliminary identification of the genus that was the most probable primary nodulating bacteria. All other NAB and Bulk Forest genera with MPS values > 0.1% were also identified and the number of NAB within 1, 2, 3, or all 4 tree age nodules was determined and compared. The MPS values of the NAB genera were analyzed for mean differences between the four different tree age nodules and the Bulk Forest soils by the Kruskal–Wallis non-parametric test, using SPSS (v.26). The number of NAB genera previously associated with any of the 9 PGP activities were also determined for each tree age nodule, ranked by MPS value, and the percent presence of genera with the potential for each PGP activity determined for each tree age nodule group.

The MPS of all of the bacterial genera were 4^th^ root transformed to account for dominant and rare taxa, as recommended by Anderson et al. [43], and converted into Bray-Curtis similarity matrices in PRIMER-E (v6) to quantify the differences between all pairs of samples [44]. These matrices were analyzed using the multivariate ANOSIM routine in the PRIMER-E package [44]–[46] to show differences between the different NAB communities, and between these and the Bulk Forest soil communities, with the number of permutations set to 9999 [46]. ANOSIM provided Global R and *p* values as the “main” test results and pairwise R and *p* values for comparisons between the different years [44]. Compositional differences and the strength of these differences identified by ANOSIM was further demonstrated using the multivariate Canonical Analysis of Principal Coordinates (CAP) ordination method [43],[47] in the PRIMER-E/PERMANOVA+ software package, with the number of permutations for the CAP analysis set to 9999, and the program allowed to select the number of PCO axes (m) that would provide the best separation between groups. This also provides a visualization of the differences between samples. The resulting CAP axis canonical correlation squared (R^2^) provided an approximation of the strength of any differences observed between group community compositions, with strong differences indicated by CAP axis R^2^ values > 0.7, moderate differences indicated by R^2^ > 0.5 to 0.69, and weak differences represented by R^2^ values < 0.5 [47].

#### 2.4.2. Differences in indicators of NAB community compositional complexity

Microbial communities in older, more established habitats (such as the older tree age root nodules) are thought to demonstrate greater community taxonomic complexity as indicated by increases in taxonomic richness, diversity, and percent taxonomic similarity, and increases in the taxonomic stability or constancy in community composition [48]–[54]. Several metrics were used to approximate the complexity level of the NAB and Bulk Forest soil bacterial communities. Margalef's richness index *d* and Shannon's diversity *H* index of the different NAB or Bulk Forest soil MPS levels were calculated in PRIMER-E. The percent taxonomic similarity of the different nodule and forest soil community compositions was determined by the multivariate SIMPER routine (similarity percentage) in PRIMER-E v.6. Lastly, the *S* (stability or constancy) index was determined for the MPS, the richness, and the diversity values, in which a greater *S* value indicates a more constant compositional makeup of a community, which is likely to occur in an older and more stable habitat [49],[50],[53]–[55]. These sets of measurements were used to indicate the level of NAB microbial community complexity [23],[48],[49],[53].

## 3. Results

### 3.1. Differences in MPS of NAB genera in the root nodules and Bulk Forest soil

**Figure 1.**
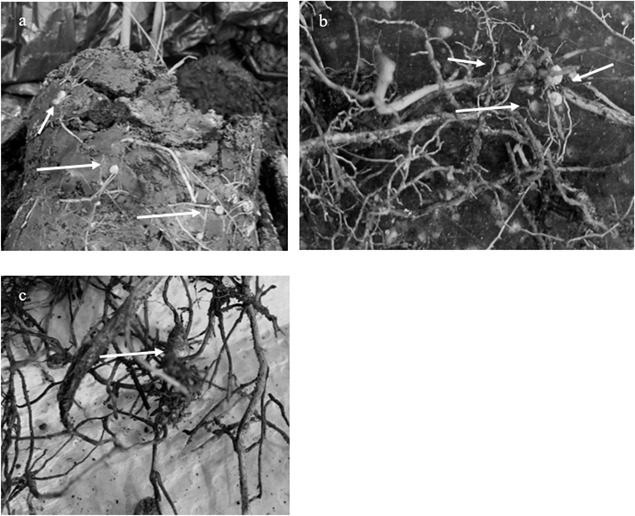
Pictures of the nodules on the roots of Inga punctata. 1a) Nodules on the roots of the 1-year-old I. punctata trees; 1b) Nodules on the roots of the 13-year-old I. punctata trees. 1c) Large nodule on the root of old growth I. punctata trees.

**Figure 2.**
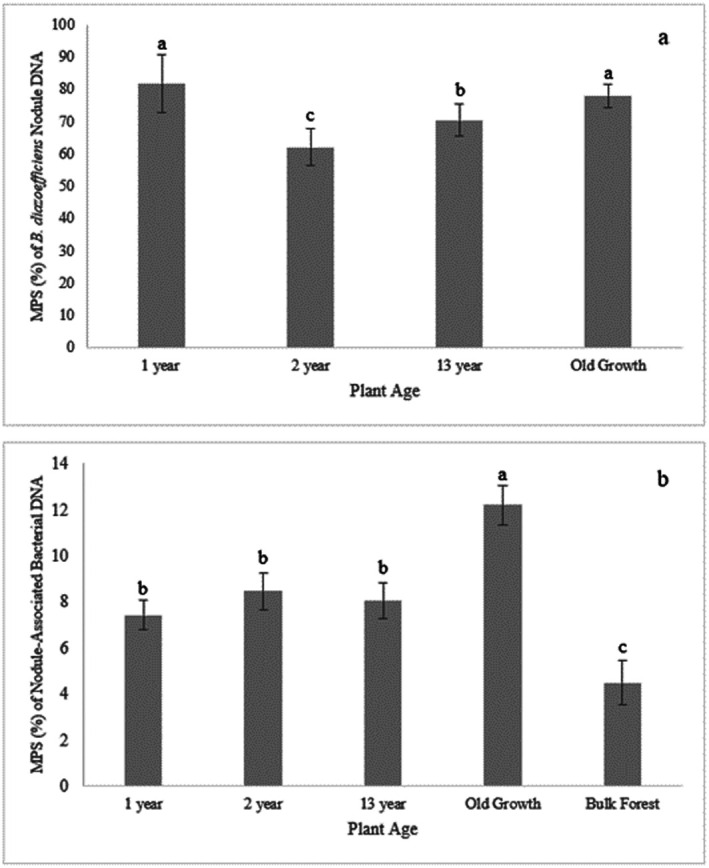
Bar charts of the mean percent of sequences (MPS as %) of *Bradyrhizobium* (possibly *diazoefficiens*) DNA and nodule associated (NAB) DNA within root nodules assessed. 2a) Bar charts of the MPS of *Bradyrhizobium* (*diazoefficiens*?) DNA within root nodules of 1-year, 2-year, 13-year, and old growth *Inga punctata* (MPS in Bulk Forest soils = 0.93, and is not shown). Different letters represent differences in mean values < 0.05 as determined by Kruskal–Wallis analyses. The MPS of *Bradyrhizobium* in the Bulk Forest soil is not shown as it was only an average of 0.18%. 2b) Bar charts of the total MPS of nodule-associated bacterial (NAB) DNA within root nodules of 1-year, 2-year, 13-year, and old growth *Inga punctata*, and Bulk Forest soil samples. Different letters represent differences in mean values, with all differences having *p* values < 0.0004, as determined by Kruskal–Wallis analyses.

Nodules were found on all ages of tree roots examined, with the minimum age of the trees where root nodules were observed was in the 1-year-old seedlings in the nursery (Figure 1a–c). The genus *Bradyrhizobium* was extremely dominant in the root nodules of all tree ages, but not in the Bulk Forest soil samples, with MPS values of 87.71% in IP1yr nodules, 63.16% in IP2yr nodules, 68.22% in the IP13yr nodules, 78.20% in the IPOG nodules, and only 0.18% in the Bulk Forest soil, suggesting *Bradyrhizobium* is most probably the primary nodule bacteria for *I. punctata* (see Table 1 and Figure 2a). There were 51 *Bradyrhizobium* OTUs within the root nodule DNA, 5 of which made up an average of 97% of the total *Bradyrhizobium* MPS values in all of the different age root nodules. There were 29 Bradyrhizobium OTUs in the Bulk Forest soil, of which 3 made up 96% of the *Bradyrhizobium* MPS in the Bulk Forest soils. A detailed BLAST search of these *Bradyrhizobium* OTUs NDA sequences showed all max scores and total scores were equal for any of the OTU, the % coverage of the reference sequences were all 100%, and all *E* scores were 0. Thus, the ranking of the most probable identity in the BLAST search result lists were based on the % identity of the DNA sequence compared to that in the database, and also the length of the read. Based on these parameters, it appears that the most probable taxon for all root nodule OTUs was *B. diazoefficiens* and for all *Bradyrhizobium* OTUs in the Bulk Forest soils, the most probable taxon was *B. kavangense* (see Table 2).

**Table 1.** The MPS (Mean %) and standard deviation (StDev) of the primary nodular bacteria (NOD) and nodule-associated bacteria (NAB) with MPS values > 0.1% in the root nodules from 1-year-old (IP1yr), 2-year-old IP2yr), 13-year-old (IP13yr), and old growth (IPOG) *Inga punctata*.

Genera in IP1yr	NOD/NAB	MPS	StDev	Genera in IP13yr	NOD/NAB	MPS	StDev
*Bradyrhizobium diazoefficiens*	NOD	85.71	9.82	*Bradyrhizobium diazoefficiens*	NOD	68.22	4.89
*Bacillus*	NAB	2.02	2.29	*Bacillus*	NAB	2.26	2.96
*Stenotrophomonas*	NAB	1.75	3.49	*Streptomyces*	NAB	1.49	2.23
*Pseudomonas*	NAB	0.83	0.93	*Pseudomonas*	NAB	0.81	1.37
*Mycobacterium*	NAB	0.67	1.25	*Rhizobium*	NAB	0.59	0.74
*Paenibacillus*	NAB	0.51	0.88	*Cupriavidus*	NAB	0.47	0.81
*Erwinia*	NAB	0.36	0.64	*Paenibacillus*	NAB	0.43	0.67
*Streptomyces*	NAB	0.33	0.73	*Mycobacterium*	NAB	0.37	0.44
*Rhizobium*	NAB	0.28	0.3	*Sphingomonas*	NAB	0.35	0.95
*Cupriavidus*	NAB	0.24	0.36	*Herbaspirillum*	NAB	0.3	0.85
*Burkholderia*	NAB	0.16	0.2	*Flavobacterium*	NAB	0.28	0.76
*Mesorhizobium*	NAB	0.13	0.24	*Klebsiella*	NAB	0.19	0.55
*Enterobacter*	NAB	0.13	0.26	*Burkholderia*	NAB	0.14	0.11
Total MPS of NAB		7.41	0.63	*Bosea*	NAB	0.12	0.16
				*Citrobacter*	NAB	0.11	0.34
				*Lysinibacillus*	NAB	0.11	0.13
				Total MPS of NAB		8.03	0.79
Genera in IP2yr	NOD/NAB	MPS	StDev	Genera in IPOG	NOD/NAB	MPS	StDev
*Bradyrhizobium diazoefficiens*	NOD	63.16	4.63	*Bradyrhizobium diazoefficiens*	NOD	78.2	3.65
*Burkholderia*	NAB	2.3	2.23	*Bacillus*	NAB	2.51	0.06
*Bacillus*	NAB	2	0.74	*Kosakonia*	NAB	2.49	1.66
*Flavobacterium*	NAB	1.39	2.49	*Mycobacterium*	NAB	2.26	0.2
*Cupriavidus*	NAB	1.11	2.1	*Pseudomonas*	NAB	1.11	0.64
*Mycobacterium*	NAB	0.37	0.32	*Enterobacter*	NAB	0.97	0.64
*Streptomyces*	NAB	0.36	0.29	*Paenibacillus*	NAB	0.61	0.18
*Paenibacillus*	NAB	0.29	0.31	*Rhizobium*	NAB	0.43	0.29
*Erwinia*	NAB	0.19	0.26	*Stenotrophomonas*	NAB	0.39	0.28
*Staphylococcus*	NAB	0.18	0.2	*Streptomyces*	NAB	0.31	0.1
*Rhizobium*	NAB	0.16	0.15	*Klebsiella*	NAB	0.21	0.15
*Caulobacter*	NAB	0.1	0.21	*Flavobacterium*	NAB	0.16	0.1
Total MPS of NAB		8.45	0.8	*Rhodococcus*	NAB	0.14	0.02
				*Bosea*	NAB	0.13	0.05
				*Nocardia*	NAB	0.13	0.2
				*Variovorax*	NAB	0.12	0.17
				*Nitrobacter*	NAB	0.11	0.09
				*Cupriavidus*	NAB	0.11	0
				Total MPS of NAB		12.2	0.87

**Table 2.** The most probable species of *Bradyrhizobium* within the *Inga punctata* root nodules and in the adjacent Bulk Forest soils. The results provided from the BLAST search performed on all *Bradyrhizobium* sequences were used to suggest the most probable species of *Bradyrhizobium* present.

Most probable *Bradyrhizobium* species in root nodules	Max score	Total score	% Coverage	*E* value	% Identity	Read length
*Bradyrhizobium diazoefficiens*	743	743	100%	0	100	1490
*Bradyrhizobium oligotrophicum*	743	732	100%	0	99.5	1432
*Bradyrhizobium americanum*	743	726	100%	0	99.5	1425
Most probable *Bradyrhizobium* species in Bulk Forest soil	Max score	Total score	% Coverage	*E* value	% Identity	Read length
*Bradyrhizobium kavangense*	743	743	100%	0	100	1414
*Bradyrhizobium subterraneum*	743	726	100%	0	99.25	1339
*Bradyrhizobium yuanmingense*	743	726	100%	0	99.25	1334

There were 7 other NAB genera identified in the nodules with the potential for nodulation (see Table 3), but as their MPS values were between 0.11% and 1.11%, as compared to the large MPS values of the presumptive *B. diazoefficiens*, these were not considered as possible primary nodulation bacteria. There were 26 other NAB genera, collectively found in the root nodules with MPS levels between 0.1% and 2.49%, with 11 of the 26 found within the IP1yr and IP2yr nodules, and 15 in the IP13yr nodules and the IPOG nodules, while 15 of the 26 genera were also found in the Bulk Forest soils with MPS levels between 0.01% and 1.14% (see Table 1). The total MPS values for these NAB genera (see Table 1 and Figure 2b) were similar in the IP1yr, IP2yr, and IP13yr nodules (~7.4%, ~8.5%, and ~8.0%, respectively), but significantly greater (*p* < 0.0001) in the IPOG nodules (~12.2%), and significantly less (*p* < 0.0004) in the Bulk Forest soil samples (~4.6%). For comparison, the total MPS for all bacterial taxa in the Bulk Forest soil samples with MPS levels > 0.1% was 87.73% (data not shown). Five of the 15 NAB genera from the Bulk Forest soils (*Bacillus*, *Flavobacterium*, *Nocardia, Rhizobium*, *and Sphingomonas*) had MPS levels greater than those associated with the root nodules (MPS range of 0.25–1.14%) and the other 10 genera had MPS levels less than (MPS range of 0.01–0.31%) that of the same genera associated with the root nodules (see Table 3).

**Figure 3.**
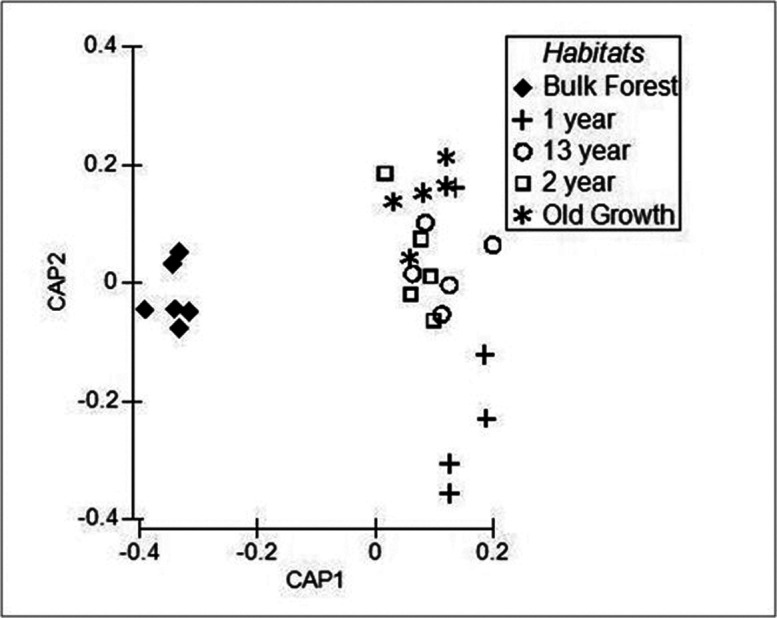
Results of the canonical analysis of principal coordinates performed on the MPS of nodule-associated bacterial (NAB) DNA within root nodules of 1-year, 2-year, 13-year, and old growth *Inga punctata*, and Bulk Forest soil samples.

The ANOSIM results further demonstrated differences in the composition of the NAB genera communities between the nodules associated with the different ages of trees, and between the Bulk Forest soils and each different tree age root nodule community (see Table 4). The IP1yr NAB community composition was different from that in the IP2yr, IP13yr, and IPOG nodules (*p* = 0.050, 0.032, and 0.028). The NAB community composition in the IP2yr was different from the IPOG community (*p* = 0.048), but not the IP13yr NAB community (*p* = 0.357), and the NAB community compositions were not different between the IP13yr and IPOG communities (*p* = 0.214). The NAB community composition in the Bulk Forest soil was different from that in the four ages of tree root nodules (all *p* values = 0.002). The CAP results (see Table 4 and Figure 3) supported the ANOSIM findings as moderate differences were found between the IP1yr and IP2yr and the IP2yr and IPOG communities (R^2^ = 0.509), strong differences were found between the IP1yr and IP13yr and the IP1yr and IPOG communities (R^2^ = 0.748 and 0.971), weak differences were found between the IP2yr and IP13yr and the IP13yr and IPOG NAB communities (R^2^ = 357 and 0.214), and strong differences were found between the Bulk Forest NAB community composition and those associated with the four tree age root nodules (R^2^ = 0.856 to 0.998).

**Table 3.** A collective list of bacteria associated with *Inga punctata* root nodules and Bulk Forest soils, and their possible functions as nodulating bacteria or plant growth promoting (PGP) bacteria. The possible PGP activities associated representatives of these genera are listed as P (protease), C (cellulase), L (lipase), Ph (phosphate solubilization), IAA (auxin production), S (siderophore production), C1 (ability to fix CO_2_ and other C1 compounds), N-Fix (nitrogen fixation), AMO (ammonium oxidation), and Nodulating (capable of forming root nodules with plants).

Genera	MPS IP1yr	MPS IP2yr	MPS IP13yr	MPS IPOG	MPS Bulk Soil	Possible plant growth promoting function
*Bacillus*	2.02	2	2.26	2.51	0.48	P, C, IAA, C1, N-Fix, AMO
*Bosea*	0	0	0.12	0.13	0	N-Fix, Nodulating
*Bradyrhizobium*	85.71	63.16	68.22	78.2	0.93	IAA, S, C1, N-Fix, AMO, Nodulating
*Burkholderia*	0.16	2.3	0.14	0	0.04	P, L, C, Ph, IAA, S, C1, N-Fix, AMO, Nodulating
*Caulobacter*	0	0.11	0	0	0.07	N-Fix, AMO
*Citrobacter*	0	0	0.11	0	0	
*Cupriavidus*	0.24	1.11	0.47	0.11	0.08	S, C1, N-Fix, Nodulating
*Enterobacter*	0.13	0	0	0.97	0.03	P, C, Ph, IAA, S, N-Fix
*Erwinia*	0.36	0.19	0	0	0	N-Fix
*Flavobacterium*	0	1.39	0.28	0.16	1.14	
*Herbaspirillum*	0	0	0.3	0	0	IAA, C1
*Klebsiella*	0	0	0.19	0.21	0	N-Fix
*Kosakonia*	0	0	0	2.49	0	Ph, IAA, S
*Lysinibacillus*	0	0	0.11	0	0	N-Fix
*Mesorhizobium*	0.13	0	0	0	0	C, Ph, IAA, C1, N-Fix, Nodulating
*Mycobacterium*	0.67	0.37	0.37	2.26	0.1	C1, N-Fix
*Nitrobacter*	0	0	0	0.11	0	C1, AMO
*Nocardia*	0	0	0	0.13	0.25	C1
*Paenibacillus*	0.51	0.29	0.43	0	0.01	P, L, C, Ph, IAA, S, N-Fix
*Pseudomonas*	0.83	0	0.81	1.11	0.31	IAA, N-Fix, AMO, Nodulating
*Rhizobium*	0.28	0.16	0.59	0.43	0.99	C, Ph, IAA, S, C1, N-Fix, Nodulating
*Rhodococcus*	0	0	0	0.14	0	IAA, N-Fix, Nodulating
*Sphingomonas*	0	0	0.35	0	0.81	Ph, IAA, N-Fix, AMO
*Staphylococcus*	0	0.18	0	0	0	
*Stenotrophomonas*	1.75	0	0	0.39	0	Ph, IAA, S, N-Fix
*Streptomyces*	0.33	0.36	1.49	0.31	0.27	C1, N-Fix, AMO
*Variovorax*	0	0	0	0.12	0.02	P, L, C, Ph, C1,
Total MPS values	7.41	8.46	8.02	11.58	4.6	

**Table 4.** Multivariate-level analysis of the differences in the composition of the nodule-associated bacteria within the root nodules of 1-year-old (IP1yr), 2-year-old (IP2yr), 13-year-old (IP13yr), and old growth (IPOG) *Inga punctata* and Bulk Forest soil samples by ANOSIM and Canonical Analysis of Principal Coordinates (CAP) assessment of the communities. The ANOSIM R and *p* values are presented for both the global and the pairwise tests, and the canonical correlation squared (R^2^) values and *p* values for the CAP are also presented. The CAP R^2^ values > 0.7 = strong differences between means, 0.50 to 0.69 = medium differences, and < 0.49 = weak differences.

	ANOSIM global test		CAP procedure
	R statistic	*p* value	Model *p* value
	0.256	0.017	0.0001
	ANOSIM paired tests	CAP procedure
Comparisons	R statistic	*p* value	R^2^ values
IP1yr, IP2yr	0.265	0.05	0.509
IP1yr, IP13yr	0.392	0.032	0.748
IP1yr, IPOG	0.444	0.028	0.971
IP2yr, IP13yr	0.032	0.357	0.132
IP2yr, IPOG	0.274	0.048	0.509
IP13yr, IPOG	0.092	0.214	0.303
Bulk Forest, IP1yr	0.856	0.002	0.971
Bulk Forest, IP2yr	0.998	0.002	0.971
Bulk Forest, IP13yr	0.965	0.002	0.971
Bulk Forest, IPOG	0.998	0.002	0.971

### 3.2. Differences in indicators of NAB community compositional complexity

There was evidence for increasing taxonomic complexity of the NAB communities occurring with increasing tree age (see Table 5). The SIMPER results showed that the percent taxonomic similarity of the NAB communities increased from the IP1yr nodules (26.17%) to that in the IP2yr and IP13yr nodules which were similar (53.85% and 50.01%, respectively), and increased again in the IPOG nodules (79.65%). Consistent with this, the *S* indices of the NAB community MPS values increased from that in the IP1yr nodules (8.73) to that in the IP2yr and IP13yr root nodules (13.65 and 13.94), and increased to a far greater level in the IPOG nodules (21.42). Margalef's richness *d* index and Shannon's diversity *H* index were relatively constant for the NAB communities in the IP1yr, IP2yr, and IP13yr nodules (*d* = 7.04, 7.31, and 7.95, and *H* = 2.37, 2.86, and 2.85, respectively) but increased significantly in the IPOG nodules (*d* = 9.33 and *H* = 3.5, respectively). However, the *S* indices for richness gradually increased from 5.28 and 5.71 in the IP1yr and IP2yr root nodules to 6.31 in the IP13yr nodules and 24.7 in the IPOG nodules, while the *S* index for the diversity *H* values increased from 6.22 in the IP1yr nodules, to 7.94 in the IP2yr nodules, to 8.91 in the IP13yr nodules, and 24.69 in the IPOG nodules. The Bulk Forest soil taxonomic community with MPS levels > 0.1% had a percent taxonomic similarity of 79.77%, richness index of 57.43, and diversity index of 5.29. The *S* index for the MPS of this community was 94.41, for the richness was 17.14, and for the diversity was 75.57. All were greater than that associated with the NAB genera community.

**Table 5.** The results of the analysis to indicate differences in complexity of the 26 NAB communities within the root nodules of 1-year-old (IP1yr), 2-year-old (IP2yr), 13-year-old (IP13yr), and old growth (IPOG) *I. punctata* root nodules, and in the Bulk Forest soil samples. Results are shown for the similarity percentage (SIMPER) analyses demonstrating the similarities in the NAB community compositions; the mean levels of Margalef's richness (*d*) and Shannon's diversity (*H*) indices with the Kruskal-Wallis analysis of these means; and the *S* indices of the MPS levels of the NAB genera and the richness and diversity of the NAB communities to show differences in the stability or constancy of the value of these metrics. Kruskal-Wallis differences in means (*p* < 0.05) in the mean values of *d* or *H* are represented by different letters. The *S* index for both *d* and *H* were also determined.

Indicators of NAB community composition stability and complexity						
	*S* Index of MPS values	SIMPER of MPS values	Mean *d*	*S* Index of *d*	Mean *H*	*S* Index of *H*
IP1yr	8.73	26.17%	7.04^a^ ± 1.33	5.28	2.37^a^ ± 0.38	6.22
IP2yr	13.65	53.85%	7.31^a^ ± 1.28	5.71	2.86^b^ ± 0.35	7.94
IP13yr	13.94	50.01%	7.95^a^ ± 1.26	6.31	2.85^b^ ± 0.32	8.91
IPOG	21.42	79.65%	9.33^b^ ± 0.38	24.7	3.50^b^ ± 0.14	24.69
Bulk Forest	4.63	89.85%	4.61^c^ ± 0.37	12.44	2.27^a, c^ ± 0.06	39.67

### 3.3. Differences in NAB genera communities with specific PGP activities

There were 23 of the 26 NAB genera previously identified in the literature to commonly have the potential to perform 1 or more of the 9 plant growth promoting (PGP) activities (see Table 6). There was also a general trend of a decreasing percentage of these NAB genera occurring in the root nodules with increasing tree age (see Table 7). There were about 56% of all IP1yr nodule NAB genera previously associated with these PGP activities, which decreased to about 41–42% in the IP2yr and IP13yr nodules, and then again to about 38% in the IPOG nodules. Six of the 9 PGP activities (cellulase production, phosphate solubilization, IAA production, siderophore production, N-fixation, CO_2_/C1 fixation) were associated with about 54% to 77% of the IP1yr nodule NAB genera, 3 of 9 PGP activities (cellulase production, N-fixation, CO_2_/C1 fixation) were associated with about 58% to 67% of the IP2yr NAB genera, 3 of the 9 activities (IAA production, N-fixation, CO_2_/C1 fixation) were associated with about 50% to 69% of the IP13yr NAB genera, and even though the IPOG nodules had the greatest MPS of NAB genera, only 2 of the 9 activities (siderophore production and CO_2_/C1 fixation) were associated with 50% and 61% of the IPOG NAB genera. The greatest decrease in genera with potential PGP activity capabilities occurred between the IP1yr and the IP2yr and IP13y nodules as the percent of the NAB genera associated with protease, lipase, cellulase, PO_4_^3−^ solubilization, and CO_2_/C1 fixation activities, and IAA and siderophore production decreased an average of 33.29%. This was followed by a 14.33% decrease in the NAB genera associated with protease, lipase, and cellulase production, PO_4_^3−^ solubilization, CO_2_/C1 fixation activities, and IAA production, and a 25% decrease in the NAB genera associated with potential siderophore production between the NAB genera in the IP13yr and IPOG nodules. The NAB genera with potential N-fixing or AMO capabilities only fluctuated between 3% to 12%.

## 4. Discussion

Members of the genus *Inga* are ecologically important leguminous trees commonly found throughout the tropical Americas [36],[56],[57]. They are thought to be important as forage resources and in enhancing the accumulation and sequestration of soil N and C, while also stimulating aboveground biomass development [1],[11],[23],[24]. However, there are gaps in the knowledge about how different species of *Inga* trees may influence the soil microbial communities and vice versa, what the taxonomic identities of the root nodule bacteria and NAB taxonomic communities of the different *Inga* species are, and if these communities differ with age or environmental condition. Preliminary work identified different species of *Bradyrhizobium* in the root nodules of some members of the genus *Inga*. For example, Leblanc et al. [11] found *B. japonicum* and *B. liaoningense* associated with *I. edulis* root nodules in South America, da Silva et al. [8] found *B. ingae* in root nodules of *I. laurina*, Aguiar et al. [25] found *B. diazoefficiens* in root nodules of *I. striata*, and Hernández-Oaxaca et al. [10] found *B. rifense* and *B centrolobii* in nodules of *I. vera*. However, to our knowledge, this current study was the first to examine the root nodules of *I. punctata* for identification of the most probable primary nodular bacteria, to characterize the total NAB community composition associated with the nodules of different ages of this tree, to determine how early the root nodules were present in young trees, and to determine if this NAB community composition changed and became more complex over time with increasing tree age.

**Table 6.** A collective list of bacteria associated with the root nodules from *Inga punctata* and plant growth promoting (PGP) activities previously associated with the genera. The PGP activities listed are for protease, lipase, and cellulase enzymatic activity, phosphate solubilization activity (PO4 Sol), auxin production (IAA or Indole-3-acetic acid), siderophore production (Sideroph), N-fixation (N-Fix), ammonium oxidation or nitrification (AMO), and CO_2_/other C1 fixations (CO_2_/C1 fix).

Genus	Protease	Lipase	Cellulase	PO_4_^3−^ Sol	IAA	Sideroph	CO_2_/C1 Fix	N-Fix	AMO	NOD
*Bacillus*	x		x		x		x	x	x	
*Bosea*								x		x
*Bradyrhizobium*					x	x	x	x	x	x
*Burkholderia*	x	x	x	x	x	x	x	x	x	x
*Caulobacter*								x	x	
*Citrobacter*										
*Cupriavidus*						x	x	x		x
*Enterobacter*	x		x	x	x	x		x		
*Erwinia*								x		
*Flavobacterium*										
*Herbaspirillum*					x		x	x		
*Klebsiella*								x		
*Kosakonia*				x	x	x				
*Lysinibacillus*								x		
*Mesorhizobium*			x	x	x		x	x		x
*Mycobacterium*							x	x		
*Nitrobacter*							x		x	
*Nocardia*							x			
*Paenibacillus*	x	x	x	x	x	x		x		
*Pseudomonas*					x			x	x	x
*Rhizobium*			x	x	x	x	x	x		x
*Rhodococcus*					x			x		x
*Sphingomonas*				x	x			x	x	
*Staphylococcus*										
*Stenotrophomonas*			x	x	x		x		
*Streptomyces*							x	x	x	
*Variovorax*	x	x	x	x			x			

The results from this work suggest that *B. diazoefficiens* is the most probable primary root nodule-forming bacteria for *I. punctata*, that it was present as early as in the nodules of 1-year-old trees (IP1yr), and that the dominance of this bacteria continues throughout the lifespan assessed for this tree. The evidence for this was that the MPS of the presumptive taxon *B. diazoefficiens* was found in all ages of root nodules from about 86% in the 1-year-old trees, to about 63% and 68% in the 2-year-old and 13-year-old trees, to about 78% in the old growth trees, while the MPS levels of the rest of the NAB genera were about 7% to 12% in the same tree root nodules. *Bradyrhizobium diazoefficiens* was previously known as one of the strains of *B. japonicum* but was recognized as a new species within the genus in 2013 [58]. Prior to that, *B. japonicum* has been identified in root nodules of tropical forest trees [12],[59],[60]. More recently, *B. diazoefficiens* has been identified in many tropical agricultural plants such as mung bean, soybean, and cow pea [61]–[65], and also in some *Inga* species [8],[12],[25], although not in *I. punctata*. Thus, it seems reasonable to suggest that the taxon presumptively identified as *B. diazoefficiens* is the nodulating bacteria for *I. punctata*. However, to confirm this, cultivation of bacteria from the macerated nodules will occur, with subsequent analysis of pure culture bacterial DNA.

**Table 7.** The percent of the nodule-associated bacterial genera with the potential for specific plant growth promoting (PGP) activities found within the root nodules of 1-year-old (IP1yr), 2-year-old (IP2yr), 13-year-old (IP13yr), and old growth (IPOG) *Inga punctata*. The PGP activities shown are for protease, lipase, and cellulase enzymatic, phosphate solubilization activity (PO4 Sol), auxin production (IAA or Indole-3-acetic acid), siderophore production (Sideroph), N-fixation (N-Fix), ammonium oxidation or nitrification (AMO), and CO_2_/other C1 fixations (CO_2_/C1 fix).

Tree Age Nodules	Protease	Lipase	Cellulase	PO4 Sol	IAA	Sideroph	N-Fix	AMO	CO_2_/C1 Fix	Mean % of all activities
IP1yr	38.46	25	76.92	53.85	69.23	61.54	69.23	38.46	69.23	55.77 ± 17.94
IP2yr	25	16.67	58.33	25	41.67	41.67	66.67	41.67	58.33	41.67 ± 17.18
IP13yr	25	23.08	43.75	31.25	50	37.5	68.75	43.75	50	41.45 ± 14.27
IPOG	22.22	16.67	38.89	27.78	44.44	50	61.11	38.89	44.44	38.27 ± 14.01

Although not as abundant as *B. diazoefficiens*, the 26 other NAB genera with MPS values > 0.1% appeared to be important root nodule microbiota, as they were present at MPS levels in the nodules of the 1-, 2-, and 13-year-old trees from about 7.5% to 8.5%, and at about 12% in the old growth tree root nodules, but only present in the Bulk Forest soils at about 4.5% MPS levels. It also appears that the NAB root nodule communities are undergoing successional development with increased taxonomic community complexity occurring with tree age as evident by the greater richness and diversity, the greater constancy (*S*) index of these two metrics, the greater *S* index of the NAB MPS values, and the greater taxonomic similarity of the NAB community all occurring as the trees age. These patterns are all indicators of an increasing complexity of the NAB community consistent with that expected to occur for any soil microbial community undergoing successional development within a habitat [23],[48]–[55].

These patterns suggest that the NAB taxa are becoming more concentrated than in the forest, and are undergoing community-like successional development to become more complex and stable, which suggests they are possibly playing important roles for nodule and/or plant growth and development. This is further supported by the identification of 26 NAB genera with the potential for PGP activities previously identified as beneficial for plant or nodule growth and development. This leads to the question of what possible roles the NAB genera and their potential PGP activities are playing in plant or root nodule functions. Although no PGP activities were definitively shown to be occurring and specifically associated with any of the NAB genera at the time of this study, the evidence in the literature potentially linking the PGP activities with different NAB genera provides an opportunity to speculate on possible associations between the potential bacterial functions and possible nodule and/or plant development and growth activities. These speculations are only preliminary suggestions meant to stimulate further analysis and testing to better understand root nodule activity, function, and influence on the nodule and plant, and are not meant to be definitive in any way.

There were 61% to 69% of the *B. diazoefficiens* root nodules that had NAB genera previously shown to have N-fixing ability, which suggests the importance of the production of NH_4_^+^ in the nodules throughout the lifespan of *I. punctata*. This supports earlier work that showed that members of the genus *Inga* prefer NH_4_^+^ over NO_3_^−^ as their inorganic N source for nodule development and activity [66]–[68]. In addition, 38% to 44% of the nodules were associated with NAB genera previously shown with the potential for the AMO activity which results in the oxidation of NH_4_^+^ into NO_2_^−^ or NO_3_^−^. It is possible that this NO_3_^−^ can be used by *B. diazoefficiens* for denitrification as this bacterium has been shown to be able to perform this specific energy transformation reaction under microoxic conditions [69]–[71], such as could occur within the root nodules.

About 41% of the *B. diazoefficiens* nodules were associated with the presence of NAB genera previously shown to be able to perform CO_2_/C1 fixation, which suggests another possible mechanism that could help *B. diazoefficiens* grow under microoxic conditions. *Bradyrhizobium diazoefficiens* is known to synthesize poly-β-hydroxybutyrate (PHB), which is used for bacterial energy storage under anaerobic or microoxic conditions, and to help bacteria to maintain N-fixing ability under such conditions [72]. Synthesis of PHB requires acetyl CoA, which can be provided by CO_2_/C1 fixation pathways [73]. As there were 41% of the nodules that had NAB genera previously associated with the potential to perform CO_2_/C1 fixation, it could suggest that using bacterial CO_2_/C1 fixation to enhance PHB production is a beneficial bacterial mechanism used by *B. diazoefficiens* to assist growth under microoxic to anaerobic conditions.

There were some PGP activities potentially linked to some of the NAB genera that were more common in the younger tree root nodules, suggesting their possible roles enhancing young root nodular or plant growth. For example, the 1-year-old tree root nodules had a greater percent of NAB genera potentially linked with protease, lipase, or cellulase activity, which may suggest a greater importance in the young tree nodules for decomposition to produce organic C used in early stages of nodular growth and development. The greater percentage of 1-year-old tree root nodules associated with potential IAA-producing NAB genera is consistent with the likely need for greater levels of production of this growth hormone to enhance growth and development of the younger trees [74]. Similarly, the greater percentage of 1-year-old nodules with NAB genera associated with the potential for siderophore production is consistent with the probable need in younger plants for iron required to perform a variety of plant physiological activities for growth enhancement [75]–[77]. Lastly, the greater percentage of 1-year-old tree root nodules with the NAB genera potentially linked to PO_4_^3−^ solubilization may indicate that the younger plants have a greater need for the PO_4_^3−^ solubilization-associated increase in phytohormone production and N-fixation activities that enhance young legume plant development [78]. Regardless of the cause, the percentage of nodules with genera associated with all of these PGP activities was greater in the 1-year-old tree root nodules, and decreased with tree age, strongly suggesting the importance of these activities in the growth and development of the nodules and/or the trees.

These results suggest that the mechanisms for growth and development assistance potentially provided by the NAB community within the root nodules of leguminous trees are much more complex than previously thought and could be linked with the potential for PGP activities associated with common NAB genera. Developing a deeper understanding of the mechanisms by which the NAB communities influence the root nodule and/or the plant will involve an assessment of these activities occurring within the root nodules at the same time as both the specific genera and the genes associated with the activities can be found.

### Summary

This study provided support that *B. diazoefficiens* may be the most probable root-nodulating bacteria of *I. punctata*, that it is dominant in the roots throughout the lifespan of *I. punctata*, and begins forming root nodules in the roots of 1-year-old trees (at the very least). The study also showed that there were NAB communities found associated with all tree age nodules with the greatest percent representation occurring associated with the IP1yr nodules. These NAB communities appear to be undergoing successional development, resulting in taxonomic communities that are becoming more complex, which is consistent with what has been observed in more advanced microbial communities following the normal successional processes seen in soil bacterial communities. The presence of these NAB communities across all ages of tree roots, and the PGP activities potentially linked with most of the NAB genera, provide evidence suggesting the importance of the NAB community for the enhanced growth and development of the nodules and *I. punctata* throughout its lifespan, but perhaps most critically in the early stages of plant growth.

Some important questions remain concerning the primary root nodule bacteria and NAB communities within *Inga* root nodules. Specifically: 1) Is it actually *B. diazoefficiens*, and can this be shown with further study? 2) Are there differences in the primary root nodule bacteria and the NAB community genera between different species of *Inga* within the same life zone and/or between life zones in the Monteverde Cloud Forest region? 3) How does the composition of these genera compare to those found within root nodules from other non-*Inga* leguminous trees in the same forests? 4) Are there seasonal-based differences in NAB community compositions, distributions, or abundances in any of these tree root nodules between wet and dry seasons, and can any such differences be used as indicators of increasing influence of a changing climate or extreme climate events in the region? 5) Is there a correlation between distances to an *Inga* species and composition of non-*Inga* legume NAB composition? Answers to these questions have implications for forest restoration in the region, as they could inform strategies on the incorporation of different species of legumes in replanting of deforested areas to enhance recovery. Also, these answers could provide information concerning potential indicators of the effects of a changing climate in the region if certain legumes and NAB compositions occur under either wetter, dryer, or warmer conditions.



## Use of AI tools declaration

The authors declare they have not used Artificial Intelligence (AI) tools in the creation of this article.

## Conflict of interest

The authors declare that they have no known competing financial interests or personal relationships that could have appeared to influence the work reported in this paper.

## Data availability

DNA data is available as stated in the Materials and methods section. Any other data will be made available upon request.

## Acknowledgements

The authors would like to thank Louisa Moreno, Monteverde Institute Lab, for her assistance and for permit acquisition, the FCC and MVI staff for access to their restoration sites, the CATIE and Patricia Leandro for the C and N metric tests, and the staff at the Rutgers University School of Medicine Molecular Biology Lab for DNA sequencing. This work was conducted through the Costa Rican government permit from CONEGEBIO MINAE # R-047-2019-OT-CONAGEBIO. This work was supported by internal funding from the Office of the Provost and Office for Undergraduate Research at Pace University, NYC, and also by the Monteverde Institute Research Department. The generated DNA sequence data is publicly available and was submitted to the NCBI-Sequence Read Archive (SRA) repository with SRA Project Code PRJNA1019742.

## Author contributions

Conceived and designed the study: WDE and DH. Performed the different collection methods: WDE, DAH, and WC. Performed the DNA sequencing methods: AL and PS. Analyzed the data: WDE, AL, and PS. Wrote the original manuscript: WDE and DAH. Read and reviewed the manuscript: WDE, DAH, WC, AL, and PS. All authors read and approved the final manuscript.
