# Supplementary material for: Use of high throughput DNA analysis to characterize the nodule-associated bacterial community from four ages of Inga punctata trees in a Costa Rican cloud forest
Source: AIMS Microbiol. 2024 Jul 23;10(3):572–95. doi: 10.3934/microbiol.2024027 (PMC11362272; doi:10.3934/microbiol.2024027)
Supplement: Supplementary file 1 [file microbiol-10-03-027-s001.pdf]

Microbiology

AIMS Microbiology, 10(3): 572–595.
DOI: 10.3934/microbiol.2024027
Received: 13 April 2024
Revised: 25 June 2024
Accepted: 03 July 2024
Published: 23 July 2024

https://www.aimspress.com/journal/microbiology

Research article

Use of high throughput DNA analysis to characterize the nodule-associated bacterial community from four ages of *Inga punctata* trees in a Costa Rican cloud forest

William D. Eaton[1,2,*], Debra A. Hamilton[3,4], Wen Chen[1], Alexander Lemenze[5], Patricia Soteropoulos[6]

[1] Biology Department, Pace University, One Pace Plaza, New York, NY 10038, U.S.A
[2] University for Peace, El Rodeo de Mora, San José, Cd Colón, Costa Rica
[3] Vermont Cooperative Fish and Wildlife Research Unit, Rubenstein School of the Environment and Natural Resources, University of Vermont, Burlington, VT 05405, U.S.A
[4] Monteverde Institute, Puntarenas, Costa Rica
[5] Molecular and Genomics Informatics Core (MaGIC), Rutgers New Jersey Medical School, 205 South Orange Avenue, Newark, NJ 07103, U.S.A
[6] The Genomics Center and Dept of Microbiology, Biochemistry and Molecular Genetics, Rutgers New Jersey Medical School, 185 South Orange Avenue, MSB F653, Newark, NJ 07103, U.S.A

* **Correspondence:** Email: weaton@pace.edu.

Supplementary

Table S1. References for the identification of bacterial genera with the potential for plant growth promoting (PGP) activities.

Number	References
1	Aserse AA, Räsänen LA, Aseffa F, et al. (2013) Diversity and sporadic symbionts and nonsymbiotic endophytic bacteria isolated from nodules of woody, shrub, and food legumes in Ethiopia. *Appl Microbiol Biot* 97: 10117–10134. https://doi.org/10.1007/s00253-013-5248-4
2	De Meyer SE, De Beuf K, Vekeman B, et al. (2015) A large diversity of non-rhizobial endophytes found in legume root nodules in Flanders (Belgium). *Soil Biol Biochem* 83: 1–11. https://doi.org/10.1016/j.soilbio.2015.01.002
3	Etesami H (2022) Root nodules of legumes: A suitable ecological niche for isolating non-rhizobial bacteria with biotechnological potential in agriculture. *Curr Res Biotechnol* 4: 78–86. https://doi.org/10.1016/j.crbiot.2022.01.003
4	Gupta P, Rani R, Usmani Z, et al. (2019) The role of plant-associated bacteria in phytoremediation of trace metals in contaminated soils, In: *New and Future Developments in Microbial Biotechnology and Bioengineering*, Amsterdam: Elsevier, 69–76. https://doi.org/10.1016/B978-0-444-64191-5.00005-5
5	Li JH, Wang ET, Chen WF, et al. (2008) Genetic diversity and potential for promotion of plant growth detected in nodule endophytic bacteria of soybean grown in Heilongjiang province of China. *Soil Biol Biochem* 40: 238–246. https://doi.org/10.1016/j.soilbio.2007.08.014
6	Liaqat F, Eltem R (2016) Identification and characterization of endophytic bacteria isolated from *in vitro* cultures of peach and pear rootstocks. *3 Biotech* 6: 120. https://doi.org/10.1007/s13205-016-0442-6
7	Martínez-Hidalgo P, Hirsch AM (2017) The nodule microbiome: N_2-fixing rhizobia do not live alone. *Phytobiomes J* 1: 70–82. https://doi.org/10.1094/PBIOMES-12-16-0019-RVW
8	Olanrewaju OS, Glick BR, Babalola OO (2017) Mechanisms of action of plant growth promoting bacteria. *World J Microbiol Biotechnol* 33: 197. https://doi.org/10.1007/s11274-017-2364-9
9	Pang J, Palmer M, Sun HJ, et al. (2021) Diversity of root nodule-associated bacteria of diverse legumes along an elevation gradient in the kunlun mountains, China. *Front Microbiol* 12: 633141. https://doi.org/10.3389/fmicb.2021.633141
10	Peix A, írez-Bahena MH, Velázquez E, et al. (2015) Bacterial associations with legumes. *Crit Rev Plant Sci* 34: 17–42. https://doi.org/10.1080/07352689.2014.897899
11	Santoyo G, Moreno-Hagelsieb G, Orozco-Mosqueda M del C, et al. (2016) Plant growth-promoting bacterial endophytes. *Microbiol Res* 183: 92–99. https://doi.org/10.1016/j.micres.2015.11.008
12	Shah A, Nazari M, Antar M, et al. (2021) PGPR in agriculture: A sustainable approach to increasing climate change resilience. *Front Sustain Food Syst* 5: 667546. https://doi.org/10.3389/fsufs.2021.667546
13	Soares R, Trejo J, Lorite MJ, et al. (2020) Diversity, phylogeny and plant growth promotion traits of nodule associated bacteria isolated from *Lotus parviflorus*. *Microorganisms* 8: 499. https://doi.org/10.3390/microorganisms8040499
14	Velázquez, E, Carro L, Flores-Félix J.D, et al. (2017) The legume nodule microbiome: A source of plant growth-promoting bacteria, In: *Probiotics and Plant Health*, Singapore: Springer, 41–70. https://doi.org/10.1007/978-981-10-3473-2_3
15	Tapia-García EY, Hernández-Trejo V, Guevara-Luna J, et al. (2020) Plant growth-promoting bacteria isolated from wild legume nodules and nodules of *Phaseolus vulgaris* L. trap plants in central and southern Mexico. *Microbiol Res* 239: 126522. https://doi.org/10.1016/j.micres.2020.126522
16	Tariq M, Hameed S, Yasmeen T, et al. (2014) Molecular characterization and identification of plant growth promoting endophytic bacteria isolated from the root nodules of pea (*Pisum sativum* L.). *World J Microbiol Biotechnol* 30: 719–725. https://doi.org/10.1007/s11274-013-1488-9

Table S2. References for identification of bacterial genera with the capability for N-Fixation or Ammonium Oxidation.

Number	References
1	Berthrong ST, Yeager CM, Gallegos-Graves LV, et al. (2014) Nitrogen fertilization has a stronger effect on soil nitrogen-fixing bacterial communities than elevated atmospheric CO_2. *Appl Environ Microbiol* 80: 3103–3112. https://doi.org/10.1128/AEM.04034-13
2	Hsu SF, Buckley DH (2009) Evidence for the functional significance of diazotroph community structure in soil. *ISME J* 3: 124–136. https://doi.org/10.1038/ismej.2008.82
3	Huang XF, Santhanam N, Badri DV, et al. (2013) Isolation and characterization of lignin-degrading bacteria from rainforest soils. *Biotechnol Bioeng* 110: 1616–1626. https://doi.org/10.1002/bit.24833
4	Isobe K, Koba K, Otsuka S, et al. (2011) Nitrification and nitrifying microbial communities in forest soils. *J For Res* 16: 351–362. https://doi.org/10.1007/s10310-011-0266-5
5	Laverman AM, Speksnijder AG, Braster M, et al. (2001) Spatiotemporal stability of an ammonia-oxidizing community in a nitrogen-saturated forest soil. *Microb Ecol* 42: 35–45. https://doi.org/10.1007/s002480000038
6	Lladó S, López-Mondéjar R, Baldrian P (2017) Forest soil bacteria: diversity, involvement in ecosystem processes, and response to global change. *Microbiol Mol Biol Rev* 81. https://doi.org/10.1128/MMBR.00063-16
7	Long X, Chen C, Xu Z, et al. (2012) Abundance and community structure of ammonia-oxidizing bacteria and archaea in a temperate forest ecosystem under ten-years elevated CO_2. *Soil Biol Biochem* 46: 163–171. https://doi.org/10.1016/j.soilbio.2011.12.013
8	Pajares S, Bohannan BJM (2016) Ecology of nitrogen fixing, nitrifying, and denitrifying microorganisms in tropical forest soils. *Front Microbiol* 7: 1045. https://doi.org/10.3389/fmicb.2016.01045
9	Reed SC, Cleveland CC, Townsend AR (2011) Functional ecology of free-living nitrogen fixation: A contemporary perspective. *Annu Rev Ecol Evol Syst* 42: 489–451.
10	Tian JH, Pourcher AM, Bouchez T, et al. (2014) Occurrence of lignin degradation genotypes and phenotypes among prokaryotes. *Appl Microbiol Biotechnol* 98: 9527–9544. https://doi.org/10.1007/s00253-014-6142-4

AIMS Press

© 2024 the Author(s), licensee AIMS Press. This is an open access article distributed under the terms of the Creative Commons Attribution License (http://creativecommons.org/licenses/by/4.0)
